# Editorial: Phytochemical changes in vegetables during post-harvest storage and processing, and implications for consumer benefits

**DOI:** 10.3389/fnut.2022.1025361

**Published:** 2022-10-13

**Authors:** Dharini Sivakumar, Yasmina Sultanbawa, Jessica L. Cooperstone, Carmit Ziv

**Affiliations:** ^1^Phytochemical Food Network, Department of Crop Sciences, Tshwane University of Technology, Pretoria, South Africa; ^2^Australian Research Council Industrial Transformation Training Centre for Uniquely Australian Foods, Queensland Alliance for Agriculture and Food Innovation, Centre for Food Science and Nutrition, The University of Queensland, Saint Lucia, QLD, Australia; ^3^College of Food, Agricultural and Environmental Sciences, The Ohio State University, Columbus, OH, United States; ^4^Department of Postharvest Science, Institute of Postharvest and Food Sciences, Agricultural Research Organization-The Volcani Center, Rishon LeTsiyon, Israel

**Keywords:** functional compounds, supply chains, post-harvest losses, phenolic, vitamins

The reduction of post-harvest losses is crucial to improving food and nutrition security by increasing food system efficiency and reducing production costs. Global food loss and waste at the retail and consumer level will be reduced by half by 2030 under Sustainable Development Goal 12.3. It also calls for reducing food losses along production and supply chains, including post-harvest losses. Developing countries suffer post-harvest losses of 15–30% due to quality standards set by retailers. The implementation of appropriate post-harvest technologies, such as cold chain management, modified atmosphere packaging, controlled atmosphere storage, and post-harvest treatments, helps in reducing post-harvest losses. For off-season use, seasonal vegetables are often preserved through post-harvest processes such as drying, dehydrating, pickling and fermentation. Processing, storage, and cooking affect phytochemicals (flavonoids, phenolic acids, anthocyanins, glucosinolates, carotenoids, and tocopherols) as well as health benefits. In fruit and vegetables, phytochemicals, otherwise known as non-nutritive compounds, enhance health due to their vital function in biological mechanisms. Growing knowledge of the chemo-preventive properties of fruits and vegetables has led to discussions about how to incorporate them into diets with modified recipes or include their functional ingredients to prevent non-communicable diseases. However, there is limited information available on the effect of post-harvest storage and processing on health-promoting phytochemicals.

Aiming to reduce food losses, increase food affordability, and improve nutrition, especially for those in need, is the goal of implementing appropriate postharvest technologies. The Research Topic aims to explore how post-harvest storage and processing techniques affect health-promoting phytochemicals in exotic and underutilized vegetables, and how this influences consumers' gut microbiome and gut health. A key element in sustaining food and nutritional security at the rural level is the identification of postharvest preservation technologies for underutilized or indigenous or traditional fruits and vegetables. Promoting sweet potato roots and leaves or other traditional vegetables and fruits as part of the diet could contribute significantly to dietary diversity.

Sixteen research articles from imminent researchers in this field are included in this Research Topic on *Phytochemical Changes in Vegetables During Post-Harvest Storage and Processing*. There are several main topics that were researched and/or reviewed, including changes in phenolic compounds and biological activities of pumpkin leaves (*Cucurbita moschata* Duchesne ex Poir.) during blanching, drying of Butternut squash (*Cucurbita moschata* Duchesne ex Poir.), steaming and drying of *Cistanche deserticola* and cooking of Sunflowers (*Helianthus annuus* L.) sprouts. The application of steam blanching to leafy vegetables in plain water was recommended to improve the antioxidant capacity for rural communities. Freeze-drying is recommended to obtain leaf powders rich in functional compounds and bioactive properties for use as functional ingredients for commercial markets. Moreover, an article on long-term frozen storage of baby mustard (*Brassica juncea* var. gemmifera) or modified atmospheric packaging enabled the retention of health-promoting substances and well suited for marketing. Additionally, ascorbic acid and antioxidative activity of lamb lettuce (*Valerianella locusta*) “Vit” salad were affected by refrigerator storage. Different cooking models affected the composition of Crocetin glycosides in saffron plants (*Crocus sativus* L.) and gardenia fruits (*Gardenia jasminoides* Ellis). Furthermore, traditional chayote leaves fermented with pineapple fruit smoothies demonstrated higher antioxidant capacity during dialysis *in vitro* digestion and the fermented product is suitable for rural communities. Furthermore, fermented Date palm fruits (*Phoenix dactilyfera* L.) are an excellent source of folates for consumers in the Middle Eastern regions. An edible herbal, *Cornus officinalis*, was shown to be effective against fibrosis after high-pressure wine-steaming (HPWS).

This special issue aims to provide more information on post-harvest processing and storage of phytochemicals and functional compounds, as well as recommendations for adapting appropriate processing and cooking methods to ensure their biological activity for consumers.

## Author contributions

All authors listed have made a substantial, direct, and intellectual contribution to the work and approved it for publication.

## Conflict of interest

The authors declare that the research was conducted in the absence of any commercial or financial relationships that could be construed as a potential conflict of interest.

## Publisher's note

All claims expressed in this article are solely those of the authors and do not necessarily represent those of their affiliated organizations, or those of the publisher, the editors and the reviewers. Any product that may be evaluated in this article, or claim that may be made by its manufacturer, is not guaranteed or endorsed by the publisher.

